# Pharmacological Protection against Ischemia-Reperfusion Injury by Regulating the Nrf2-Keap1-ARE Signaling Pathway

**DOI:** 10.3390/antiox10060823

**Published:** 2021-05-21

**Authors:** Bercis Imge Ucar, Gulberk Ucar, Sarmistha Saha, Brigitta Buttari, Elisabetta Profumo, Luciano Saso

**Affiliations:** 1 Department of Biochemistry, Faculty of Medicine, Hacettepe University, 06100 Ankara, Turkey; 2 Department of Biochemistry, Faculty of Pharmacy, Hacettepe University, 06100 Ankara, Turkey; gulberk@hacettepe.edu.tr; 3 Department of Cardiovascular, Endocrine-Metabolic Diseases, and Aging, Italian National Institute of Health, 00161 Rome, Italy; sarmistha_pharmacol@yahoo.com (S.S.); brigitta.buttari@iss.it (B.B.); elisabetta.profumo@iss.it (E.P.); 4 Department of Physiology and Pharmacology “Vittorio Erspamer” Sapienza University, 00161 Rome, Italy; luciano.saso@uniroma1.it

**Keywords:** Nrf2/Keap1/ARE signaling pathway, ischemia-reperfusion injury, Nrf2 activators

## Abstract

Ischemia/reperfusion (I/R) injury is associated with substantial clinical implications, including a wide range of organs such as the brain, kidneys, lungs, heart, and many others. I/R injury (IRI) occurs due to the tissue injury following the reestablishment of blood supply to ischemic tissues, leading to enhanced aseptic inflammation and stimulation of oxidative stress via reactive oxygen and nitrogen species (ROS/RNS). Since ROS causes membrane lipids’ peroxidation, triggers loss of membrane integrity, denaturation of proteins, DNA damage, and cell death, oxidative stress plays a critical part in I/R pathogenesis. Therefore, ROS regulation could be a promising therapeutic strategy for IRI. In this context, Nrf2 (NF-E2-related factor 2) is a transcription factor that regulates the expression of several factors involved in the cellular defense against oxidative stress and inflammation, including heme oxygenase-1 (HO-1). Numerous studies have shown the potential role of the Nrf2/HO-1 pathway in IRI; thus, we will review the molecular aspects of Nrf2/Kelch-like ECH-associated protein 1 (Keap1)/antioxidant response element (ARE) signaling pathway in I/R, and we will also highlight the recent insights into targeting this pathway as a promising therapeutic strategy for preventing IRI.

## 1. Introduction

### 1.1. Ischemia/Reperfusion Injury (IRI)

IRI injury is a sequential catastrophic event with high morbidity and mortality, usually seen after thoracoabdominal surgeries, cardiopulmonary bypass, hemorrhagic, traumatic, or septic shock, severe burns, or after transplantation operations [1] characterized by loss of blood flow, causing hypoxia and lack of nutrition (ischemia phase) followed by restoration of the blood supply (reperfusion phase), which often results in systemic inflammatory response syndrome (SIRS) and multiple organ dysfunction syndrome (MODS) [2,3,4,5,6]. Interruption of the blood flow causes reduced oxygen-dependent-ATP production, impaired ion-pump activities, hypoxanthine and lactate accumulation (acidosis), intracellular calcium overload, which triggers reactive oxygen species (ROS) production. Reintroducing oxygen to the ischemic tissue contributes to the formation of oxidant-dependent pro-inflammatory mediators, increase in calcium overload in the cell. It also activates calpains leading to programmed cell death, ROS production from previously sequestered hypoxanthine consequent to reoxygenation, thereby results in a positive feedback loop by opening the mitochondrial permeability transition pores (mPTP) to produce more ROS. Eventually, excess amounts of ROS result in oxidative stress and oxidase proteins, lipids, and DNA [6,7].

### 1.2. Oxidative Stress

Oxidative stress is a pathological process caused by the imbalance between redox systems in favor of the oxidant systems. This imbalance usually originates either from the excessive production of ROS or the diminished ROS scavenging capacity [3,4,5]. ROS and reactive nitrogen species (RNS) can be sub-grouped into free radicals like superoxide (O_2_^•−^), oxygen, hydroxyl, and peroxyl radicals, nitric oxide and nitrogen dioxide, and non-radicals such as hydrogen peroxide (H_2_O_2_), hypochlorous acid, singlet oxygen, nitrous acid, nitroxyl anion, lipid peroxides’ aldehydes, and peroxynitrite [3,4,5,8,9,10,11]. These can be introduced from exogenous sources like environmental pollutants [8,9,10], ultraviolet irradiation [9,10], carcinogens [9,10], and cigarette smoke [9,10], produced on purpose for physiological signal transduction of cells such as inflammation, integrin recruitment, growth factor signaling (for instance, correct interaction between insulin, epidermal growth factor, platelet-derived growth factor, and their receptors), adhesion to the extracellular matrix, or can be a metabolic by-product [3,4,5,8,9,10,11]. The xanthine oxidase system, NADPH oxidase system, mitochondrial electron transport chain, uncoupled nitric oxide synthase system, and non-enzymatic sources (like hemoglobin and myoglobin) generate oxidative stress [5,8]. 

### 1.3. Pro-Inflammatory Features of IRI

Tissue destruction can be seen under excessive ROS exposure via lipid peroxidation in the cell membrane and organelle lipids, DNA oxidation, activation of matrix metalloproteinases, induced osmotic cell lysis, increased mitochondrial permeability, or via indirect mechanisms including free radical formation by the interaction with molecular oxygen, NO, free iron, or fatty acids or free iron. These mechanisms enhance the formation of oxidant-dependent pro-inflammatory mediators and upregulation of cytokine/chemokine and adhesion molecule expression during reperfusion [7]. Indeed, although the mechanism of IRI is a form of pathogen-free inflammation, it displays a series of inflammatory reactions resembling infectious processes such as inducing cytokine and chemokine production with infiltration of immune system cells [3,4]. After reperfusion, the reciprocity between the innate and adaptive immune cells is critical for tissue damage induction. Neutrophils and monocytes become activated by many damage-associated molecular patterns (DAMPs) released by necrotic cells, which in turn polarize T lymphocytes toward the Th1 pro-inflammatory phenotype. The primary immune response expands tissue-associated damage by inducing NLRP3-inflammasome, TLR9, and the NET formation [12,13].

Consequently, the myeloid differentiation primary response gene 88 (MyD88) and nuclear factor-κB (NF-κB) pathways are activated, followed by initiation of the inflammatory mediator release [14], which amplifies the aforementioned immune cells. NLRP3 inflammasome activation also generates caspase-mediated cell death [15]. From a wound-healing perspective, after the inflammatory phase dominated by Th1 polarized cells, a second, anti-inflammatory reparative phase leading to wound healing and scar formation comes later. Cytokines have a crucial role in tissue repair. IL-6, IL-10, transforming growth factor-beta (TGF-β), and a subpopulation of T-lymphocytes known as ‘T Regulatory cells’ (Treg) have all been associated with suppressing the pro-inflammatory response and steering the immune system towards repair and resolution following I/R [16,17]. Apoptotic neutrophils induce an M2 phenotype in infiltrated macrophages upon their phagocytosis, inhibiting the macrophage pro-inflammatory tissue-damaging response and leading them to produce IL-10 and TGF-β [18,19]. Notably, IL-10 may serve to dampen both Th1 and Th2 inflammation, thus inhibiting I/R-derived damage, as well as excessive tissue scarring during the reparative phase. Accumulation of immune and inflammatory cells causes ROS to pile up and fibrosis by inducing fibroproliferation and abnormal collagen accumulation, as well as inflammation-mediated tissue destruction. Additionally, endothelial swelling and glycocalyx degradation in I/R lead to loss of intercellular contact between endothelial cells, consequently causing various vascular abnormalities, including elevated vascular permeability and leakage, dysregulated vasodilation and vasoconstriction, dysfunction of the endothelial cell barrier, inflamed endothelium, along with activation of complement and coagulation systems [3].

Other manifestations of I/R with similar pro-inflammatory features include cell death via necrosis or programmed cell death (apoptosis and autophagy). In normal conditions, autophagy recycles damage proteins and organelles after lysosomal degradation as a cytoprotective mechanism. However, ROS accumulation during I/R surpasses autophagic clearance capacity and eventually leads to cell death [3,6]. It has already been known that the disproportionate generation of ROS is the crucial mechanism underlying IRI [2,3,4,5,6,7]. This excessive ROS and RNS accumulation are kept down by water- and liposoluble antioxidants like glutathione and liposoluble vitamins, as well as antioxidant enzymes. Enzymatic detoxification of ROS includes dismutation of O_2_^•−^ by superoxide dismutase (SOD), generating the formation of H_2_O_2_, which can be handled either by catalase or by glutathione peroxidase (GPx) and glutathione reductase [8,11]. 

### 1.4. Nuclear Factor (Erythroid-Derived 2)-Related Factor 2 (Nrf2)/ Kelch-Like ECH-Associated Protein 1 (Keap1)/ Antioxidant Response Element (ARE) 

**Figure 1:**
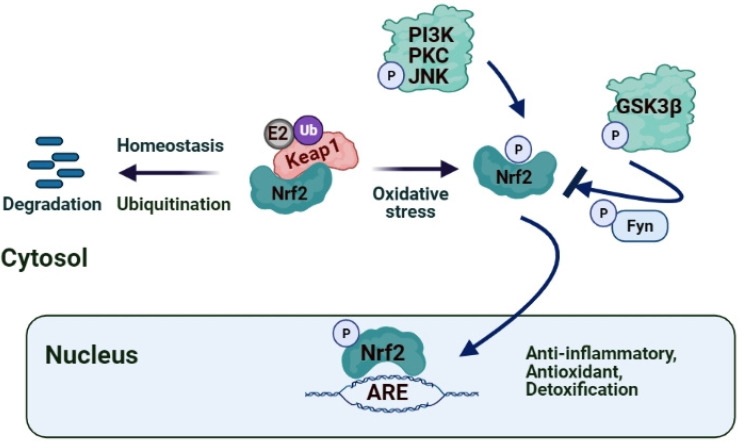
Under normal conditions, Keap1 homodimerizes and forms Keap1-Cul3-E3 ligase complex, which facilitates Nrf2 ubiquitination and degradation. During I/R injury, Nrf2 is released from this complex and translocates into the nucleus without and binds with ARE to trigger the transcription of endogenous protective genes. Additionally, during this condition, PI3K, JNK, and PKC activate Nrf2 via phosphorylation, whereas GSK-3β inhibits Nrf2 activation through Fyn kinase activation. (ARE: Antioxidant Response Element, Keap1: Kelch-like ECH-associated protein 1, GSK3β: Glycogen synthase kinase-3β Nrf2: Nuclear factor (erythroid-derived 2)-related factor 2, P: phosphate, PI3K: Phosphoinositide 3-kinase, UbE2: Ubiquitin-conjugated E2 enzyme).

**Figure 2:**
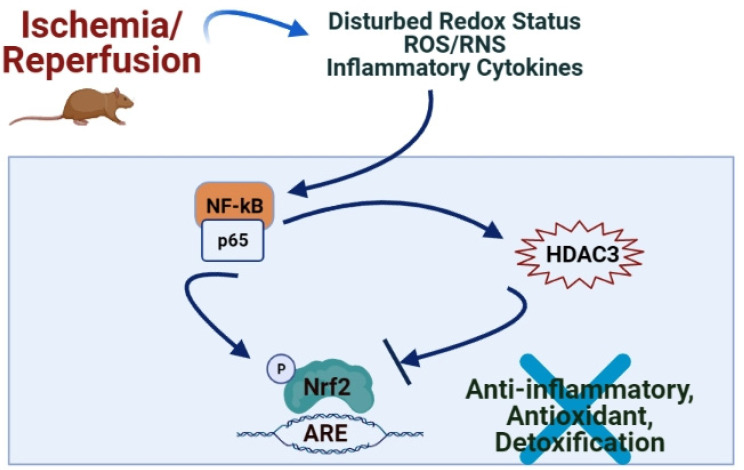
During I/R injury, activation of NFκB-p65 occurs, which in cooperation with HDAC3, negatively regulates Nrf2-ARE signaling and promotes oxidative stress-induced cell death. (ARE: Antioxidant Response Element, HDAC3: Histone Deacetylase 3, Nrf2: Nuclear factor (erythroid-derived 2)-related factor 2).

The Nrf2/Keap1/ARE pathway regulates these aforementioned ARE-responsive antioxidant proteins [11,20,21,22,23,24,25,26]. The essence of Nrf2 signaling also lies in Nrf2-Notch crosstalk, which influences the defense systems and cytoprotection against endogenous and exogenous stressors and enhances the maintenance of cellular homeostasis and tissue organization through actions on cell proliferation kinetics and cell differentiation [27]. Besides these, Nrf2 exerts anti-inflammatory and anticancer activities by regulating its multiple downstream cytoprotective genes, thereby plays a vital role in cell survival [28]. It has also been shown that Nrf2 redirects glucose and glutamine into anabolic pathways, especially under the sustained activation of PI3K-Akt signaling, which augments the nuclear accumulation of Nrf2 and enables Nrf2 to promote metabolic activities that support cell proliferation and enhances cytoprotection [29] (Figure 1). Recent studies also showed an inter-relation between the Nrf2/ARE system and the expression of inflammatory mediators, NF-κB pathway, and macrophage metabolism [30] (Figure 2). Hence, after discovering Nrf2 and its key regulator Keap1 5 years apart [1994 (Yuet Wai Kan’s lab researchers) and 1999 (Itoh et al.), respectively] [9], more studies focused on their prominent role in signaling and redox homeostasis in the last two decades [21] and regulation of inflammation [30].

In this review, we will focus on the functions of the Nrf2/Keap1/ARE signaling pathway in IRI and the structure and regulation of this pathway’s elements, along with the potential relevance of this pathway as a promising therapeutic target in IRI.

## 2. Structural and Functional Properties of Nrf2

Nrf2, encoded by the NFE2L2 gene in humans and classified under the cap “n” collar (CNC) subfamily of basic-region leucine zipper (bZIP) transcription factors along with other five (NF-E2, Nrf1, Nrf3, Bach1, and Bach2), is present in various cell types. It comprises 605-amino-acid-long protein with seven highly conserved Nrf2-ECH domains (Neh1-7), which serve as a different functional region [20,21,24,25,26]. Among these, Neh1 is the DNA-binding domain, and the CNC–bZIP is essential in recognizing DNA to promote heterodimerization. Next, Neh2 domain, which consists of DLG *(low affinity)* and ETGE *(high affinity)* motifs for the interaction with Keap1. Neh3 is responsible for the ARE activation. Neh4 and Neh5 are responsible for binding with different “cAMP response element-binding” (CREB) proteins and activate transcription. Neh6 regulates the protein stability by phosphorylation of serine residues or ubiquitination by b-TrCP. Neh7 is accountable for the RXRα (retinoic acid receptor-alpha) interaction and the disruption of the binding between CBP (CREB-binding protein) and the Neh4 and Neh5, as well as inhibition of the ARE sequence transcription. Nrf2 stays in its inactive form within cells via Keap1 unless there is a disturbance in redox homeostasis. Activated Nrf2 accumulates in the cell nucleus and binds to ARE on DNA and upregulates related genes’ transcription to reduce production or induce elimination of ROS [22,25,26,31] (Figure S1).

## 3. Regulation of Nrf2

The Nrf2/Keap1/ARE pathway is a crucial mechanism to defend the cell against the chemical and oxidative stress caused by endogenous (i.e., electrophiles, oxidants) or exogenous (i.e., xenobiotics) factors by activating antioxidant genes in order to restore the redox balance [24,26,32]. Thus, the regulation of this pathway is critical in terms of physiological cellular homeostasis. As previously mentioned, under normal cellular conditions, Nrf2 is the leading regulator of the diverse range of cytoprotective genes [22,33]. Still, ARE-responsive genes are required only at basal levels so that Keap1 suppresses nuclear translocation of Nrf2 to keep its transcriptional activity low [31] (Figure S1). 

Keap1 is a cysteine-rich (27 cysteines), cytoplasmic, actin cytoskeleton-associated adapter zinc-metalloprotein of the Cul3/Rbx1 complex. It consists of five domains, and Keap1/Cul3 homodimerization is regulated by the N-terminal portion of the intervening region with the BTB domain. In contrast, Nrf2/Keap1 dimerization is regulated by the Kelch domain’s interaction and the C-terminal region of Keap1 with ETGE and DLG motifs of Nrf2’s Neh2 domain [22,24,33,34] (Figure S1). Keap1 serves as a cytoplasmic factor that interacts with the Neh2 domain and forms the Keap1-Nrf2 complex to sequester Nrf2 in the cytoplasm and initiates degradation of Nrf2 by ubiquitination and proteasomal degradation unless there is a stress stimulus around [22,33,34]. The N-terminal of the Neh2 domain comprises lysine and serine residues. These seven lysine residues serve as a target for KEAP1-dependent ubiquitination, whereas the serine residue (ser40) is essential for nuclear translocation of Nrf2 following Keap1/Nrf2 dissociation by phosphorylation of ser40 by protein kinase C [22,26,31,34,35,36,37] (Figure 1).

Another mechanism concerning the separation of Nrf2 and Keap1 is modifying the several cysteines in Keap1 [38,39]. Cysteine code hypothesis is based on the conformational changes in the Keap1 by cysteine modifications in the presence of electrophiles and oxidants leading to inhibition of polyubiquitination of Nrf2 via disturbance of the interaction between Neh2 (particularly DLG motif) and Kelch domains [34].

As it is mentioned above, the Nrf2/Keap1 pathway is the master regulator of expression of the genes related to antioxidant proteins, phase I and II electrophile detoxification enzymes. It inhibits inflammation, recognizes the DNA damage, mediates glutathione homeostasis, proteasome function, and is also responsible for the transportation of toxic solutes and regulating the expression of detoxifying enzymes. These are accomplished by the interaction of Nrf2 with the antioxidant response element (ARE) [22,33]. 

Once the Nrf2/Keap1 complex is disrupted, Nrf2 dimerization with small Maf (sMaf) protein occurs to form Nrf2-sMaf heterodimers. sMaf proteins have a similar bZIP domain as Nrf2, which makes them Nrf-2-competitors binding to ARE. Nrf2-sMaf heterodimer formation ensures binding of Nrf2 to ARE due to the higher concentration of this heterodimer in the nucleus than the sMaf homodimers’ formation. Nrf2 binds to cis-regulatory ARE sequence (5′-RTGACnnnGC-3′) to control the basal and inducible expression of antioxidant and detoxifying genes under stressed conditions caused by xenobiotics, metals, and UV irradiation [20,31,33,40] (Figure S1). 

Besides, ARE sequences are present in the regulatory regions of genes such as glutamate-cysteine ligase, NAD(P)H quinone oxidoreductase 1 (NQO1), heme-oxygenase-1 (HO-1), sulfiredoxin1 (SRXN1), heme-oxygenase (HO-1), glutathione S-transferase (GST), multidrug resistance-associated proteins (MRPs), and UDP-glucuronosyltransferase (UGT) [22]. The HO isoenzymes are the rate-limiting enzymes in the degradation of heme to carbon monoxide, bilirubin, and iron, which have a significant role in modulating physiological functions. Three HO isoforms (HO-1, -2, and -3) have been identified. HO-1 is involved in maintaining the balance of antioxidants and oxidants in the process of cell damage, and it is the most induced antioxidant enzyme induced by Nrf2. Under physiological conditions, Nrf2 is sequestered in the cytosol by Keap1 and is targeted for proteasomal degradation. In the presence of ROS, Nrf2 is released from Keap1 and then translocates into the nucleus, activating the transcription of target genes, including HO-1. Overexpression of HO-1 was shown to protect the organs from IRI via anti-inflammatory and anti-apoptotic effects. Pharmacologic-induced upregulation of HO-1 protects cells and organs, whereas HO-1 inhibition may abolish the effect and aggravate the damage [41,42].

## 4. Role of Nrf2/Keap1/ARE Pathway in IRI

In both normal and stressed conditions, redox balance can be maintained mainly at the transcription level via Nrf2/Keap1/ARE signaling. This pathway controls over 250 genes involved in antioxidant systems and detoxification, and cytoprotection [9,26,33,43,44].

Nrf2 primarily regulates the transcription of the related genes of detoxifying and antioxidant enzymes, which function as ROS scavengers and electrophile neutralizers (i.e., superoxide dismutase (SOD), glutathione peroxidase (GPx), glutathione reductase (GR), catalase), as well as induces glutathione synthase (indirectly). Besides, the activated Nrf-2/heme oxygenase-1 (HO-1) axis causes heme to degrade into bilirubin, an antioxidant substance [23,26]. Nrf2 cooperates with NF-κB signaling pathways to maintain the physiological homeostasis of cellular redox status and to regulate the cellular response to stress and inflammation [30] (Figure 1). Accumulating evidence also suggests that Nrf2 counteracts the NF-κB-driven inflammatory response by competing with transcription co-activator cAMP response element (CREB) binding protein (CBP) [45,46,47]. However, numerous studies showed that different pathological sources of exogenous or endogenous stress often activate both NF-κB and Nrf2-ARE signaling [30].

### 4.1. Disease-Specific Effects of Nrf2/Keap1/ARE Pathway within the IRI Concept

The Nrf2/Keap1/ARE signaling pathway has been established as the primary cellular defense mechanism against oxidative stress both in physiological conditions and in a wide array of disease models of various systems [33].

#### 4.1.1. Central Nervous System (CNS)

As previously reviewed in the literature, although the reestablishment of blood flow resolves acute stroke during CNS ischemia, oxidative stress causes tissue damage and the prevention of complete recovery of cerebral functions during the reperfusion period [48]. Hence, several studies emphasized the importance of the antioxidant regulatory role of Nrf2 during different stages of cerebral ischemia, with their notable findings on experimental I/R models in different parts of the brain. In focal cerebral I/R models in which Nrf2 is upregulated at the mRNA and protein levels with increased Nrf2-binding activity to the ARE and accumulation of Nrf2 in the nucleus, the target antioxidant proteins of Nrf2 were also upregulated. Besides, immunohistochemical investigations in these studies revealed that Nrf2 levels in the peri-infarct areas are also significantly elevated along with Nrf2’s target antioxidative proteins thioredoxin, glutathione, and HO-1 [47,48,49,50]. 

#### 4.1.2. Urinary System

IRI of the kidneys caused by irregular renal blood flow following renal transplantation is a serious issue in which pro-inflammatory cytokines and chemokines, ROS, and mitochondrial dysfunction are involved [51]. Here, Nrf2 is shown to be a significant regulator of the impaired redox balance in renal I/R [52,53,54]. In one of the studies, the expression of Nrf2-target genes was found to be induced instantly after IRI (and consequent oxidative stress) and returned to basal levels within 24 h [53]. Additionally, in Keap1 gene-deleted mice, tubular epithelial cells were found to be responsible for the Nrf2-mediated renoprotection under oxidative stress [55,56,57], and the severity of renal IRI is accentuated both in the murine model [58] and the Nrf2-knockout mice model [59]. Furthermore, T cell-specific activation of Nrf2 is reported to be renoprotective in I/R-induced acute kidney injury in mice [60]. 

#### 4.1.3. Gastrointestinal System

Intestines are among the most sensitive organs to I/R, and intestinal IRI can be seen in various scenarios following obstruction or strangulation of the intestines, thromboembolic incidents, shock, and intestinal transplantation. Intestinal IRI results in inflammation and loss of the intestinal barrier followed by bacterial translocation [1,4,61].

The protective effect of Lipoxin A4 was demonstrated in a rat model of intestinal IRI, where lipoxin A4 alleviated the IRIvia Keap1/Nrf2 pathway activation [62]. Similarly, in an experimental study on a rat model of liver transplantation-induced intestinal injury, the HO-1 elevation through the Nrf2/Keap1 pathway ameliorated the intestinal mucosa injury and tight junction dysfunction [63].

#### 4.1.4. Respiratory System

End-stage respiratory diseases require lung transplantation, and IRI is a significant issue in primary graft dysfunction. Lung IRI (LIRI) also can be seen during cardiopulmonary bypass surgery, trauma, pulmonary thromboembolism, or cardiac arrest [64]. It is recently reported that the activated Keap1/Nrf2/HO-1 pathway may protect the lungs from IRI via restraining alveolar macrophage pyroptosis in LIRI by tuning the redox balance in favor of the antioxidant system [64]. Another study noted that Nrf2 and related antioxidant enzyme activation boost the recovery from IRI in cold-preserved and transplanted lungs [65]. Preconditioning lung allografts via inhaled hydrogen gas in the donor’s lungs before transplantation induces heme oxygenase (HO)-1, which is transcriptionally regulated by the Nrf2/ARE pathway and protects the organs from the incoming IRI [66]. It is also indicated that the activated Nrf2/ARE signaling pathway plays a part in the propofol-induced reduction of LIRI [67]. Moreover, Nrf2 plays a protective role in I/R-induced acute lung injury by increasing the expression of HO-1 and SLC7A11(a gene encoding a cystine/glutamate xCT transporter) and regulating ferroptosis [68].

#### 4.1.5. Hepatobiliary System

Hepatic IRI often occurs due to hemorrhagic shock or surgical interventions such as hepatic resections and liver transplantation. Several studies proposed that hepatic IRI is mitigated by Nrf2/Keap1 complex via Keap1 signaling and induction of Nrf-2 to increase HO-1 expression, leading to boosted hepatocyte autophagy and subsequently discarded impaired mitochondria, and reduced ROS production [69,70,71]. Another experimental study suggested that vagal nerve stimulation attenuates IRI in the liver through elevation of Nrf2/HO-1 proteins with antioxidant and anti-inflammatory properties, as well as prohibitive properties on apoptosis [70]. Furthermore, it has been demonstrated that Itaconate/Immune-Responsive Gene 1 pathway has a protective effect on hepatic IRI because it activates Nrf2-mediated antioxidative reactions in hepatocytes [71].

#### 4.1.6. Cardiovascular System

Acute myocardial ischemia is caused by the occlusion of the coronary arteries, which restrains the heart’s blood flow, followed by cardiac muscle tissues’ necrosis [72,73]. Routine therapeutic options for myocardial ischemia usually include percutaneous or open interventions and thrombolytic medicine to restore blood flow which initiates the reperfusion period [74]. An experimental study investigating the antioxidant and anti-inflammatory effects of tanshinone IIA in rat cardiomyocytes during oxidative stress injury suggested that these functions originate from the Nrf2 pathway [75]. Similarly, some other studies using various experimental and clinical data concluded that the up-regulation of Nrf2 mitigates the escalation of hemodynamic stress and prevents the resultant heart failure; thus, it could be a potential therapeutic target for cardiovascular diseases caused by IRI [73,76,77]. Additionally, several studies proposed that epidermal growth factor (EGF) has a vital role in the Nrf2 pathway, as activation of EGF receptor (EGFR) enables the nuclear translocation of Nrf2 by inhibiting Keap1 to carry out the antioxidant activities following transcription of NQO1 and HO-1 [78]. EGF loses its antioxidative and antiapoptotic features if Nrf2 is silenced [79]. Furthermore, in an earlier study, prostaglandin D_2_ is reported to have cardioprotective properties during IRI through Nrf2 activation via the PGF2α receptor (FP) [80]. 

## 5. Nrf2/Keap1/ARE Pathway as a Potential Target in IRI Therapy

### 5.1. Cardiac IRI

Myocardial IRI (MIRI) is a severe pathophysiological condition associated with complex mechanisms, including oxidative stress, cell apoptosis, and inflammatory responses [5,6,7]. Overproduction of ROS activates various molecular cascades of apoptosis [8,9]. Furthermore, inhibition of ROS production or free radicals scavenging is suggested as potential therapeutic strategies to attenuate IRI [2,81]. Nrf2 signaling is associated with the cleavage process during MIRI, as Nrf2 disassociates from Keap1, translocates to the nucleus where it binds to AREs, and regulates target genes’ expressions during oxidative stress [82,83]. The Nrf2 signaling pathway reportedly protects against anoxia/reoxygenation-induced apoptosis [73]. 

Zhang et al. [84] suggested that melatonin may protect H9c2 cells against IRI by reducing apoptosis and oxidative stress, mediated via activation of the Nrf2 signaling pathway. The protective effect of atorvastatin is generated via the inhibition of neutrophil infiltration, TNF-α production, and activation of the Nrf2/ARE pathway, leading to upregulation of HO-1, a sensitive and reliable indicator of cellular oxidative stress [85]. L-carnitine can reduce MIRI via activating the Nrf2/HO-1 signaling pathway and reducing oxidative stress and apoptosis in cardiomyocytes. Zhao et al. [86] reported the significant escalation of the Nrf2/HO-1 signaling pathway in myocardial tissue in L-carnitine-injected rats, which indicates that L-carnitine activates the Nrf2/HO-1 signaling pathway and reduces oxidative stress in cardiomyocytes. The bardoxolone derivative, DH404, showed protective effects against infarct expansion and remodeling post-MI in rats by re-coupling eNOS and increasing the functional interaction of Grx1 eNOS, and activates Nrf2 [87]. 

Plant polyphenols, such as flavonoids, chalcones, triterpenes, and proanthocyanidins, have cardioprotective, antiapoptotic, and anti-ischemic properties, which cause a decrease in the infarct size, arrhythmia score, and improvement in cardiac stunning primarily via the release of NO, the activation of the Nrf2 pathway and endogenous antioxidant defense system [88]. Resveratrol, the polyphenolic compound present in red grapes and wine, ameliorates cardiac dysfunction induced by MI/R by activating the Nrf2/ARE pathway and increases the expression of Nrf2 by inducing SIRT1 or inhibiting GSK3β, thus alleviating myocardial oxidative stress, and thereby improving IRI [89,90]. It was suggested that through the activation of PI3K/Akt/p38 MAPK, H_2_O_2_ preconditioning alleviate the caspase-3 activity, increases the expression of Bcl2, and leads to the translocation of Nrf2 into the nucleus, which selectively increases the expression and activity of antioxidant enzymes to prevent MIRI-induced apoptosis [76]. Triptolide, the bioactive component present in *Tripterygium wilfordii* Hook F, reportedly reduces I/R-induced myocardial infarction, inflammation, oxidative stress and improves the cardiac function in rats via its effect on the Nrf2/HO-1 defense pathway [91]. Aloin, the major bioactive anthraquinone of the Aloe species, also diminishes I/R-induced oxidative stress injury and inflammatory response in cardiomyocytes by activating the Nrf2-HO-1 signaling [92]. Recent studies indicated that garlic (*Allium sativum*) and its constituents, such as Allicin and Diallyl sulfide, were reported to activate Nrf2 and increase HO-1 and NQO1 expression through ERK/p38 signaling pathway activation, thus playing a protective role in the adaptation of diabetic cardiomyopathy [93]. Another polyphenolic compound, luteolin, contained in vegetables, was shown to protect the diabetic heart against IRI by enhancing eNOS-mediated S-nitrosylation of Keap1, with subsequent upregulation of Nrf2 and the Nrf2-related antioxidative signaling pathway [94]. New curcumin analog, 14p, decreased oxidative stress and reduced MIRI via increasing Nrf2 expression [83]. Kaempferide (3,5,7-trihydroxy-4-methoxy flavone) inhibits Nrf2 and cleaved caspase-3 signaling pathways through a PI3K/Akt/GSK 3β-dependent mechanism and attenuates I/R-induced myocardial injury [95] (Figure 1). Simultaneously, Butin, a plant dietary flavonoid, protects against I/R-induced ROS-mediated apoptosis by upregulating the AMPK/Akt/GSK-3β pathway and activates Nrf2-regulated antioxidant enzymes in diabetic cardiomyocytes exposed to I/R [96]. Crocin, the active component of saffron, suppresses I/R-induced cardiomyocyte apoptosis via ER stress inhibition by modulating the miR-34a/Sirt1/Nrf2 signaling pathway [97]. Hyperoside, a flavonoid isolated from Rhododendron ponticum L., was suggested to have a protective effect on cardiac IRI by inhibiting ER stress and activating the Nrf2 signaling pathway in an ischemia/reperfusion animal model [98]. Soybean isoflavones are shown to activate Nrf2-mediated antioxidant responses in a dose-dependent way and alleviate ischemic cardiomyopathy [99]. 

### 5.2. Hepatic IRI

The role of Nrf2 in hepatic I/R was identified by several studies showing that the Keap1–Nrf2 complex may alleviate oxidative injury [100]. Nfr2 was proposed as a fundamental transcription factor involved in diminishing hepatic IRI since the interaction between Keap1 and Nrf2 is disrupted in I/R induced stress conditions, and Nrf2 translocates into the nucleus promoting the transcription of cytoprotective and antioxidant genes such as heme oxygenase 1 (HO-1) [70]. The triterpenoid CDDO-imidazoline (CDDO-Im), a synthetic oleanane triterpenoid and potent activator of the Nrf2 pathway, was found to ameliorate liver IRI in mice by inducing the expression of Nrf2 target gene HO-1, which lead to enhanced autophagy in hepatocytes, and to reduced ROS production and inflammatory responses [69]. Ibrahim et al. [101] studied the protective effects of dimethyl fumarate, which is an FDA approved immunomodulatory drug that induces the anti-inflammatory stress protein HO-1, and curcumin, a polyphenol found in the rhizome of the herb *Curcuma longa*, against acute hepatic injury induced by I/R as a combination therapy focusing on the Nrf2/HO-l signaling pathway. They concluded that the combination of these two drugs has a synergistic hepatoprotective effect against hepatic-induced I/R via activating Nrf2/HO-1 pathways. 15-Deoxy-Δ12,14-prostaglandin J2 has been proposed to alleviate hepatic IRI in mice via inducing the Nrf2 pathway, which leads to inhibition of ROS generation, apoptosis, and autophagy [102]. Propofol post-conditioning was postulated to ameliorate hepatic IRI by reducing ROS levels through the enhanced expression of Nrf2. Pretreatment with Sulforaphane, an Nrf2 agonist, reduced apoptosis and oncosis via activating the Nrf2/ ARE pathway, ameliorated oxidative stress, and attenuated hepatic IRI [103]. Telluric acid, a tellurium-based compound, was reported to ameliorate hepatic I/R-induced injury in rats by affecting TLR4, Nrf2, and PI3K/Akt signaling pathways [104]. The transmembrane G protein-coupled bile acid receptor (TGR5) plays a vital role in regulating energy homeostasis, glucose metabolism and possesses protective activity for inflammation by suppressing the NF-κB signaling pathway. Zhuang et al. [105] recently demonstrated that TGR5 reduced hepatocellular apoptosis and inhibited inflammatory response by regulating Keap1-Nrf2 signaling pathways, suggesting that the TGR5 molecule may appear as a novel and potential therapeutic target for preventing hepatic IRI. The IRG1/Itaconate pathway was recently suggested to activate Nrf2 in Hepatocytes and improve I/R induced hepatic injury. 4-IO, a new itaconate derivative, was found to increase the expression and nuclear translocation of Nrf2 and up-regulation of its downstream protective pathways (HO-1 and NQO1) during hepatic injury both in mouse and human hepatocytes [71]. 

Brahma-related gene 1 (Brg1), a catalytic subunit of SWI2/SNF2-like chromatin remodeling complexes, is suggested to activate the Nrf2/HO-1 pathway during hepatic I/R. Recently, Ge et al. [106] showed that hepatic Brg1 overexpression during reperfusion enhanced Nrf2-mediated inducible expression of HO-1 during hepatic I/R, causing an increase in antioxidant ability and the recovery of liver IRI. Perindopril, an ACE inhibitor, was recently presented to be beneficial in hepatic IRI by suppressing hepatic oxidative and inflammatory stresses through the modulation of NF-κB-p65/TLR-4, JAK1/STAT-3, Nrf-2, and PI3K/Akt/mTOR signaling pathways [107]. Sevoflurane, an inhalation anesthetic widely used in clinical practice, has been observed to induce oxidative stress and affect cognitive functions [108]. More recently, Ma et al. [109] postulated that sevoflurane promoted Nrf2 entry into the nucleus, upregulated the downstream target gene HO-1, activated the Nrf2 gene pathway, and protected the liver from IRI. 

Fisetin (3,3′,4′,7-tetrahydroxyflavone), a flavonoid found in various fruits [110]; Gastrodin, a bioactive compound extracted from the traditional Chinese herbal agent [111]; Myricetin, a flavonoid glycoside extracted from the fruits, leaves, and branches of *Myrica cerifera* [112] and CDDO-Im [69] were reported to alleviate liver damage and oxidative stress in liver I/R through the Nrf2/HO-1 signaling pathway, suggesting that these compounds may be considered as targeted drugs for liver IRI treatment.

### 5.3. Intestinal IRI 

Redox imbalance, nitric oxide, leukocyte adhesion molecule P-selectin, NF-κB, TNF-α, IL-1β, cellular adhesion molecules, and inducible nitric oxide synthase (iNOS) play a central role in intestinal IRI [7]. Interleukin-1 receptor antagonist (IL-1Ra) possesses a protective effect on intestinal IRI by anti-inflammatory, antioxidative, and antiapoptotic mechanisms via the activation of Nrf2/HO-1 and MAPKs pathways, suggesting it as a new therapeutic drug for the improvement of intestinal IRI [113]. Irisin, a new peptide, promotes heat production, ameliorates obesity, and regulates glucose homeostasis to improve I/R-induced intestinal inflammatory response, reduce oxidative stress, and inhibits apoptosis, which could be associated with the Nrf2 pathway activation [114]. Dimethyl fumarate activates antioxidant pathways, induces Nrf2/HO-1, GSK-3β Wnt/β-catenin signaling pathways, and improves I/R-induced intestinal inflammatory response [115]. Salvianolic acid A ameliorated oxidation inhibited the release of pro-inflammatory cytokines, and alleviated apoptosis in I/R-induced injury through the regulation of Nrf2/ HO-1 pathways [116]. Ginsenoside Rb1, a major active saponin isolated from ginseng, attenuates intestinal ischemia/reperfusion-induced inflammation and oxidative stress via activation of the PI3K/Akt/Nrf2 signaling pathway [117]. Higenamine (1-[(4-hydroxyphenyl) methyl]-1,2,3,4-tetrahydroisoquinoline-6,7-diol) is an active ingredient of *Aconiti Lateralis Radix Praeparata* with anti-inflammatory properties, and it has been shown to regulate the Nrf2-HO-1-High mobility group box 1 protein (Hmgb1) axis and attenuate intestinal IRI in mice which appears as a novel therapy to protect intestinal IRI [118].

### 5.4. Cerebral IRI

Ischemic stroke is a worldwide debilitating clinical disorder and one of the most common causes of death. Reoxygenation of the ischemic brain causes IRI. Excessive ROS production is related to cerebral IRI in stroke. Recent studies are focused on the therapeutic potential of targeting the Nrf2/HO-1 pathway in brain injury after ischemic stroke [119]. 11-Keto-β-boswellic acid, the pentacyclic triterpenoid compound of Boswellia serrata resin, increased Nrf2 HO-1 expression in a middle cerebral artery occlusion model of ischemic injury and protected the brain from oxygen and glucose deprivation-induced oxidative insult [120]. Hu et al. [121] postulated that Panax notoginseng saponins (PNS) activate Nrf2 antioxidant signaling depending on the PI3K/Akt pathway and protect against oxygen-glucose deprivation/reperfusion-induced BBB dysfunction. Z-ligustilide, the main lipophilic component of *Radix Angelica sinensis*, reportedly activates the Nrf2/HO-1 pathway, induces Nrf2 nuclear translocation, and upregulates HO-1 expression in a time- and concentration-dependent manner [122]. RS9, a novel Nrf2 activator obtained from bardoxolone methyl (BARD), showed neuroprotective effects against cerebral I/R via targeting NF-κB and Nrf2-ARE pathway and attenuated neuroinflammation [123]. Nomilin, a triterpenoid extracted from citrus fruits, exhibits many biological activities, including immunomodulatory activity. Nomilin ameliorates blood–brain barrier disruption in the middle cerebral artery occlusion rat model, possibly via alleviating the loss of the tight junction proteins ZO-1 and occludin-5 and increasing the expressions of Nrf2 and NQO1 [124]. Monomethyl fumarate, a pharmacologically active metabolite of dimethyl fumarate, has been reported to reduce cerebral edema by attenuating oxidative stress and inflammation [125]. Singh et al. [126] recently demonstrated the potential of monomethyl fumarate’s activity as an Nrf2 activator in the peri-infarct cortex. Resveratrol, a very well-known stilbene, has been shown to possess neuroprotective effects primarily by reducing oxidative stress and mitochondrial dysfunction [127]. Pterostilbene from blueberries was reported to exhibit neuroprotective effects via Nrf2 activation [128]. Xu et al. [129] recently compared the neuroprotective effects of pinosylvin, pterostilbene, pinostilbene, and 4-methoxy-trans-stilbene using oxygen and glucose depletion/reperfusion model in PC12 cells. They indicated that pinosylvin has a significant neuroprotective effect by inducing PINK1/Parkin mediated mitophagy and also activating the Nrf2 pathway to ameliorate oxidative stress-induced mitochondrial dysfunction. Further, 2-Cyano-3,12-Dioxooleana-1,9-Dien-28-Oic acid (CDDO), and its derivatives exhibit anti-inflammatory and antioxidant activities. In addition, 6 CDDO-ethyl amide (CDDO-EA) increased HO-1 expression, and primed microglial polarization toward M2 phenotype under inflammatory stimulation in BV2 microglial cells, suggesting that CDDO-EA confers neuroprotection against cerebral ischemic injury [130]. 

### 5.5. Renal IRI 

Acute kidney injury (AKI) caused by ischemia-reperfusion also constitutes a major clinical issue. RTA-408, a new oleanane triterpenoid compound, induces Nrf2 and downstream antioxidant gene expression, protects the kidney from renal I/R by activating the Nrf2 antioxidant system and increasing the Nrf2 nucleus translocation and subsequently upregulating of downstream antioxidant genes, including GSH biosynthesis [131]. Hyperglycemia-induced oxidative stress is involved in impaired SIRT1/Nrf2/HO-1 signaling and ischemia AKI in diabetes. Melatonin was proposed to ameliorate ischemia AKI in diabetes by improving the SIRT1/Nrf2/HO-1 signaling, suggesting that the SIRT1/Nrf2/HO-1 pathway can be a new target in decreasing the oxidative stress in diabetic IRI [132]. Synthetic triterpenoids such as CDDO (2-cyano-3,12-dioxoolean-1,9-dien-28-oic acid) and its methyl (CDDO-Me, bardoxolone methyl) and imidazolide (CDDO-Im) derivatives have been shown to activate Nrf2-mediated antioxidant activity in several diseases, including chronic kidney disease. CDDO-Im treatment upregulated Nrf2 target gene activation, induced the expressions of cytoprotective genes, including GCLC, HO-1, and NQO1, resulted in significant improvements in renal function and increased survival [131]. The naturally occurring antioxidant, nordihydroguaiaretic acid, prevents renal damage and apoptosis by inducing Nrf2 translocation in renal IRI [133].

Table 1 summarizes the potential compounds which may be protective against I/R injury in several organs by inducing the Nrf2 pathway.

## 6. Conclusions

I/R plays a significant role in the pathogenesis of numerous diseases, including liver, heart, kidney, brain, intestine, testis, eye, extremities, etc. (Table 1). Ischemia induces transcriptional reprogramming leading to post-translational activation of hypoxia and inflammatory signaling cascades, while reperfusion causes severe damage by induction of inflammatory responses. Oxidative stress generated by excessive production of oxygen free radicals plays an essential role in I/R mediated tissue damage. Nrf2 is a key molecule responsible for the proper regulation of the antioxidant system and cytoprotective genes. Emerging evidence indicates that Nrf2 induces the expression of antioxidant enzymes and plays a pivotal role in the protection against oxidative stress. Its activity is regulated by Keap1, an adaptor subunit of cullin 3-based E3 ubiquitin ligase. Nrf2 translocates from the cytoplasm to the nucleus under the stress condition, thus activating the expression of multiple target genes encoding antioxidant and drug-metabolizing enzymes and transporters and heme and iron metabolic enzymes.

Many experimental studies have revealed that the protective role of the Nrf2 pathway in IRI I/R enhances Nrf2 dissociation from Keap1, facilitating Nrf2 translocation to the nucleus, binding to the ARE, and activating antioxidant and anti-inflammatory genes. In the present review, we focused on the Nrf2-ARE pathway as a target against oxidative stress-induced IRI and reported data on different bioactive compounds that demonstrated to be able to activate this pathway. Recent studies indicated that Nrf2 activation might play a vital role in preventing IRI. The modulation of the Nrf2-Keap1 pathway by combining different chemicals appears as a promising target for preventing and treating organ damages caused by IRI. In conclusion, combining different bioactive compounds, by promoting Nrf2-mediated protection against oxidative stress- and inflammation-derived injury, may represent new therapeutic strategies against IRI.

## Figures and Tables

**Table 1:** An overview of potential compounds with a protective effect on I/R injury by inducing the Nrf2 pathway.

Compound	Related System	References
Atorvastatin	Cardiovascular system	[85]
L-carnitine	Cardiovascular system	[86]
DH404	Cardiovascular system	[87]
Plant polyphenols (flavonoids, chalcones, triterpenes, and proanthocyanidins)	Cardiovascular system	[88]
Resveratrol	Cardiovascular system	[89,90]
Triptolide	Cardiovascular system	[91]
Aloin	Cardiovascular system	[92]
Allicin	Cardiovascular system	[93]
Diallyl sulfide	Cardiovascular system	[93]
Luteolin	Cardiovascular system	[94]
14p (a curcumin analog)	Cardiovascular system	[83]
Melatonin	Cardiovascular system Urinary system	[84] [132]
Kaempferide	Cardiovascular system	[95]
Butin	Cardiovascular system	[96]
Crocin	Cardiovascular system	[97]
Hyperoside	Cardiovascular system	[98]
Soybean isoflavones	Cardiovascular system	[99]
CDDO-imidazoline	Hepatobiliary system Urinary system	[69] [131]
Dimethyl fumarate	Hepatobiliary system	[101]
Curcumin	Hepatobiliary system	[101]
15-Deoxy-Δ12,14-prostaglandin J2	Hepatobiliary system	[102]
Sulforaphane	Hepatobiliary system	[103]
Telluric acid	Hepatobiliary system	[104]
4-IO (an itaconate derivative)	Hepatobiliary system	[71]
Perindopril	Hepatobiliary system	[107]
Sevoflurane	Hepatobiliary system	[108,109]
Fisetin (3,3′,4′,7-tetrahydroxyflavone)	Hepatobiliary system	[110]
Gastrodin	Hepatobiliary system	[111]
Myricetin	Hepatobiliary system	[112]
Interleukin-1 receptor antagonist (IL-1Ra)	Gastrointestinal system	[113]
Irisin	Gastrointestinal system	[114]
Dimethyl fumarate	Gastrointestinal system	[115]
Salvianolic acid A	Gastrointestinal system	[116]
Ginsenoside Rb1	Gastrointestinal system	[117]
Higenamine (1-[(4-hydroxyphenyl) methyl]-1,2,3,4-tetrahydroisoquinoline-6,7-diol)	Gastrointestinal system	[118]
11-Keto-β-boswellic acid	Central nervous system	[120]
Panax notoginseng saponins	Central nervous system	[121]
Z-ligustilide	Central nervous system	[122]
RS9 (a novel Nrf2 activator obtained from bardoxolone methyl)	Central nervous system	[123]
Nomilin	Central nervous system	[124]
Monomethyl fumarate	Central nervous system	[125,126]
Resveratrol	Central nervous system	[127]
Pterostilbene	Central nervous system	[128]
Pinosylvin	Central nervous system	[129]
6 CDDO-ethyl amide (CDDO-EA)	Central nervous system	[130]
RTA-408 (a new oleanane triterpenoid compound)	Urinary system	[131]
Nordihydroguaiaretic acid	Urinary system	[133]

CDDO: 2-Cyano-3,12-Dioxooleana-1,9-Dien-28-Oic acid.

## Supplementary Materials

The following are available online at https://www.mdpi.com/article/10.3390/antiox10060823/s1, Figure S1 the structure and functions of Nrf2.
