# Supplementary material for: Pharmacological Protection against Ischemia-Reperfusion Injury by Regulating the Nrf2-Keap1-ARE Signaling Pathway
Source: Antioxidants (Basel). 2021 May 21;10(6):823. doi: 10.3390/antiox10060823 (PMC8224095; doi:10.3390/antiox10060823)
Supplement: Supplementary file 1 [file antioxidants-10-00823-s001.zip › Figure S1 (antioxidants-1194634-supplementary).pdf]

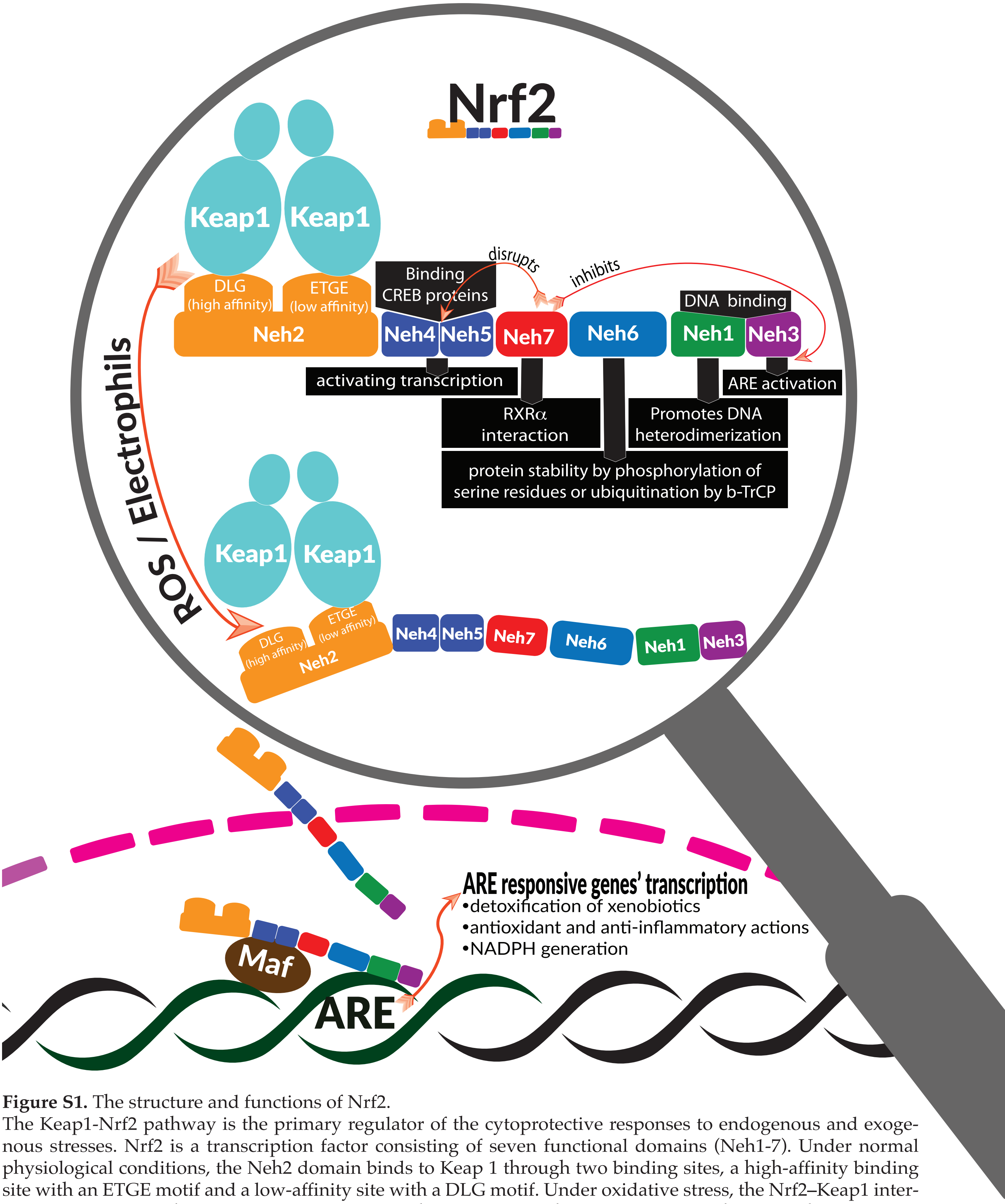

Figure S1. The structure and functions of Nrf2.

The Keap1-Nrf2 pathway is the primary regulator of the cytoprotective responses to endogenous and exogenous stresses. Nrf2 is a transcription factor consisting of seven functional domains (Neh1-7). Under normal physiological conditions, the Neh2 domain binds to Keap 1 through two binding sites, a high-affinity binding site with an ETGE motif and a low-affinity site with a DLG motif. Under oxidative stress, the Nrf2–Keap1 interaction is resolved. The free Nrf2 translocates to the nucleus and heterodimerizes with one of the small Maf proteins, and activates the ARE (antioxidant response element) site. Disruption of the interaction between Nrf2 and Keap1 triggers the activation of the Nrf2 pathway; Nrf2/Maf complex activates the ARE-dependent gene expression of a series of antioxidative and cytoprotective proteins and plays a significant role through its anti-inflammatory, antioxidant, detoxification, autophagy, and proteasome actions. (**ARE:** Antioxidant Response Element, **CREB protein:** cAMP-response element-binding protein, **b-TrCP:** Beta-transducin repeat-containing proteins, **Keap1:** Kelch-like ECH-associated protein 1, **Maf:** musculoaponeurotic fibrosarcoma oncogene homolog, **NADPH:** Nicotinamide adenine dinucleotide phosphate, **Neh:** Nrf2-ECH homology domain **Nrf2:** Nuclear factor (erythroid-derived 2)-related factor 2, **RXR***α***:** Retinoid X receptor alpha).
